# Use of online health information to manage children’s health care: a prospective study investigating parental decisions

**DOI:** 10.1186/s12913-015-0793-4

**Published:** 2015-04-02

**Authors:** Anne M Walsh, Kyra Hamilton, Katherine M White, Melissa K Hyde

**Affiliations:** School of Nursing, Queensland University of Technology, Victoria Park Road, Kelvin Grove, 4059 Queensland, Australia; School of Applied Psychology, Griffith University, Messines Ridge Road, Mt Gravatt, 4122 Queensland, Australia; School of Psychology and Counselling, Queensland University of Technology, Victoria Park Road, Kelvin Grove, 4059 Queensland, Australia; Menzies Health Institute Queensland, Griffith University, Gold Coast Campus, Southport, 4222 Queensland, Australia

**Keywords:** Child online health information, Online information seeking behaviour, Theory of planned behaviour, Beliefs, Parental decision making

## Abstract

**Background:**

The use of the internet to access information is rapidly increasing; however, the quality of health information provided on various online sites is questionable. We aimed to examine the underlying factors that guide parents’ decisions to use online information to manage their child’s health care, a behaviour which has not yet been explored systematically.

**Methods:**

Parents (N = 391) completed a questionnaire assessing the standard theory of planned behaviour (TPB) measures of attitude, subjective norm, perceived behavioural control (PBC), and intention as well as the underlying TPB belief-based items (i.e., behavioural, normative, and control beliefs) in addition to a measure of perceived risk and demographic variables. Two months later, consenting parents completed a follow-up telephone questionnaire which assessed the decisions they had made regarding their use of online information to manage their child’s health care during the previous 2 months.

**Results:**

We found support for the TPB constructs of attitude, subjective norm, and PBC as well as the additional construct of perceived risk in predicting parents’ intentions to use online information to manage their child’s health care, with further support found for intentions, but not PBC, in predicting parents’ behaviour. The results of the TPB belief-based analyses also revealed important information about the critical beliefs that guide parents’ decisions to engage in this child health management behaviour.

**Conclusions:**

This theory-based investigation to understand parents’ motivations and online information-seeking behaviour is key to developing recommendations and policies to guide more appropriate help-seeking actions among parents.

**Electronic supplementary material:**

The online version of this article (doi:10.1186/s12913-015-0793-4) contains supplementary material, which is available to authorized users.

## Background

Parents use various sources to gather information about their child’s health care, including the internet [1,2]. In 2008, an Australian study found that 43% of parents sought child health information on the internet [1], with higher rates reported in other developed countries [3]. Reasons for using online health information include perceptions of feeling rushed when seeking information and not receiving general lifestyle advice from doctors (i.e., health information is focused on the immediate care only) [4]. Parents regard online health information as more up-to-date, quicker and easier to access than offline information, and often trust the information provided [5]. Online information is used to source specific information about their child’s health issues [e.g., diabetes [4], impending surgery [6-8], or parenting infants [9]. However, parents can be selective in seeking information. For example, those with a child diagnosed with cancer found online information scary, preferring to receive cancer-related information from a trusted health professional [10]. Thus, reports of parents both trusting and mistrusting online child health information coexist in the literature.

The quality of online health information is questionable. There is little control over the timeliness of updates and inaccurate information is being reported (see, for example, papers on reports of school health information [11] and emergency health information [12]). A systematic review of websites offering advice on acute otitis media treatments identified 41% of sites still recommending antibiotics while only 31% recommended the new guideline of ‘watch and wait’ [13]. Furthermore, despite readily available evidence-based information about the safety of childhood immunisations, parent online discussion forums still purport the potential harm from child immunisations [14,15].

Considering the questionable quality of available online information, parent-reported actions following accessing online health information are concerning. In Australia in 2006, 43% of parents reported diagnosing and 33% treating their child’s health condition using online information and of concern was that 18% altered their child’s health management to align with online information [16]. Most Australian households have internet access (83% in 2012-2013) and, of these, 96% were households with children under the age of 15 years [17]. Despite this saturation of internet access, there are limited reports of a corresponding increase in, or monitoring of, the quality of available online health material. Internet-based interventions to support, guide and change health behaviours are now commonplace (e.g., [18-20]); however, few studies theoretically explore the underlying factors influencing parents’ decision-making in using online child health information.

It is timely, therefore, to identify the factors influencing parents’ child health information-seeking behaviour. Previous research in this area has been limited in approaches focusing mostly on demographic differences between users and nonusers [21], sites explored, and factors influencing information-seeking for specific types of information including asthma [22], cancer [10], specific surgery [8], and eHealth literacy [23]. More recent research has started to understand the processes guiding online information-seeking, suggesting intention to search for information, for example vaccination information [24], is influenced by attitudes and perceived social norms. However, there is a dearth of research systematically exploring the underlying factors influencing parents’ decisions to use online child health information in general (i.e., not condition-specific) nor have many previous studies drawn from well validated theoretical frameworks reflecting established decision-making processes. This innovative study, therefore, applied a sound theoretical approach, the Theory of Planned Behaviour (TPB; [25]), to understand parents’ decision-making in managing their children’s health. Errors in parents’ judgement when managing their children’s health can have grave consequences. Theory-based investigations to understand parents’ motivations and information-seeking behaviour are, therefore, important to develop recommendations and inform policies to guide appropriate on-line help-seeking actions among parents.

The TPB is a sound model of behavioural decision making which specifies intentions as predicting behaviour (measured prospectively; in the case of the current study at 2 months follow up to allow a reasonable period of time in which parents may be faced with a health-related issue for their child). Intentions, in turn, are predicted by attitude (positive/negative evaluations of the behaviour), subjective norm (perceived pressure from others to perform the behaviour), and perceived behavioural control (PBC, perceived ease/difficulty of performing the behaviour; also believed to influence behaviour directly) [25]. Attitude, subjective norm, and PBC are informed by underlying behavioural beliefs (costs and benefits), normative beliefs (others’ approval/disapproval) and control beliefs (barriers and motivators), respectively [25]. A number of studies have utilised the knowledge of these underlying beliefs to increase our understanding of people’s decision making (e.g., [26-30]). No previous study has, however, documented the critical beliefs underlying parents’ decisions to use online information to manage their child’s health care.

Despite the strong support demonstrated for the TPB [31], a large proportion of the variance remains unexplained leading researchers to propose additional variables to help explain people’s decision making. Given the potentially unreliable information presented on online sites [11,12], errors in judgement may harm one’s health [32]. Accordingly, there may be risks associated with using online information to manage a child’s health care. Risk perceptions have been explored in other TPB-based studies where it was found to predict people’s willingness to engage in risky behaviour [33-35]. Given the added value of risk perceptions to the TPB and that using online information to manage a child’s health care may be considered risky behaviour, investigating this construct in this context seems warranted. In particular, the role of risk should be examined in this context in conjunction with the more established decision-making factors to assess its impact on people’s online help-seeking intentions when also taking into account constructs such as people’s attitudes.

This study was part of a larger project investigating influences on parents’ online child health information-seeking behaviours [36]. This paper reports an examination of the factors guiding parents’ decisions to use child health information from the internet to manage their child’s health care. As per the TPB [25], we hypothesised that attitude, subjective norm, and PBC would predict parents’ intentions (Hypothesis 1) and that intention and PBC would directly influence their behaviour (Hypothesis 2). For the additional factor of risk perception, we expected that parents who perceive greater risk in using online health information to manage their child’s health care would have weaker intentions to do so (Hypothesis 3). In addition, to identify the critical beliefs guiding parents’ intentions, we expected that significant correlations between the behavioural, normative, and control beliefs and intentions would be observed and that some of the significant key beliefs would independently predict parents’ intentions (Hypothesis 4).

## Methods

### Participants

Participants were Australian parents who were current internet users and had at least one child aged 6 months to 10 years. An upper age limit of 10 years was used as children aged 10 years and older may search online for health information themselves [37]. Parents were recruited via online advertising techniques (e.g., forums on parenting websites, university and parenting email newsletters).

### Design and procedure

Ethical clearance was obtained by Queensland University of Technology’s Human Research Ethics Committee (approval #0800000840) and a prospective design with two waves of data collection was adopted. The main questionnaire comprised the standard TPB measures (i.e., attitude, subjective norm, PBC, intention) as well as the underlying TPB belief-based items (i.e., behavioural, normative, and control beliefs) in addition to a measure of perceived risk and demographic variables (Additional file 1). Two months later, consenting participants completed a follow-up telephone questionnaire which assessed the decisions they had made regarding their use of online information to manage their child’s health care during the previous 2 months (Additional file 2).

### Measures

For this study, the target behaviour was using “child health information from the internet to manage my child’s health care” in the next 2 months. The 2 month interval was considered a reasonable period of time in which parents may be faced with a health-related issue for their child. For example, given that children tend to have between four and six febrile illnesses a year during the first two years of life [38,39], the 2 month follow-up period seemed appropriate, particularly in consideration of the need to maintain engagement of parents within the study. A definition for child health information was included: “When we talk about child health information we mean any information that you may find online that helps you to make a decision about how to care for your child’s health. This information may include, but is not limited to, information about the appropriate age to introduce solids, management of an existing condition such as asthma or dietary intolerances, vaccination, an upcoming medical procedure or test, advice about how to identify, diagnose, or treat a rash or a fever, etc.”.

#### Pilot study

An elicitation study was conducted with 23 Australian parents (2 fathers, 21 mothers; *M*_*age*_ = 35.35 years, *SD* = 4.31, Range = 29-45 years) who were current internet users. Individual and group interviews were used to identify the most commonly occurring behavioural, normative, and control beliefs, and other experiences related to the use of the internet to access child health information. Consistent with the specifications of the belief-basis of the TPB, the interview guide comprised open-ended questions as outlined by Fishbein and Ajzen [40]. The open-ended questions were designed to elicit beliefs without instilling any preconceived notions. Thematic content analysis was undertaken to identify the most common responses to each of the TPB belief-based questions, with responses coded according to the questions tapping into the behavioural (advantages/disadvantages), normative (important others approving/disapproving), and control (barriers/facilitators) beliefs [41].

### Main study questionnaire

#### Intention

One item assessed intention (“I intend to use [target behaviour]”, scored *strongly disagree* [1] to *strongly agree* [7]).

#### Attitude and behavioural beliefs

One item assessed attitude (“Using [target behaviour] would be good”, scored *strongly disagree* [1] to *strongly agree* [7]).

The belief-based measures of attitude were assessed by 19 behavioural beliefs elicited from the pilot study. Parents rated how likely the 11 benefits and 8 costs would result if they were to perform the target behaviour, scored *extremely unlikely* [1] to *extremely likely* [7]).

#### Subjective norm and normative beliefs

One item measured subjective norm (“Most people who are important to me would support/approve of me using [target behaviour]”, scored *strongly disagree* [1] to *strongly agree* [7]).

The belief-based measures of subjective norm were assessed by the seven normative beliefs elicited from the pilot study. Parents rated how likely the seven referents would approve of/support their performance of the target behaviour, scored *extremely unlikely* [1] to *extremely likely* [7]).

#### Perceived behavioural control and control beliefs

One item measured PBC (“It is mostly up to me whether I use [target behaviour]”, scored *strongly disagree* [1] to *strongly agree* [7]).

The belief-based measures of PBC were assessed by 7 control beliefs elicited from the pilot study. Parents rated how likely the four barriers and three motivators would prevent or motivate them, respectively, to perform the target behaviour, scored *extremely unlikely* [1] to *extremely likely* [7]).

#### Perceived risk

Perceived risk was assessed by one item (“It would be risky for me to use [target behaviour]”, scored *strongly disagree* [1] to *strongly agree* [7]).

### Follow up questionaire

#### Behaviour

Parents’ behaviour was measured with two items, “In the past 2 months I have used [target behaviour]”, scored *strongly disagree* [1] to *strongly agree* [7]; “In the past 2 months how often did you use [target behaviour]?”, scored *never* [1] to *always* [7]). The two items were averaged to form the behaviour measure and the items were significantly correlated, *r*(181) = .74, *p* < .001.

### Statistical analysis

A descriptive analysis of means, standard deviations, and bivariate correlations was performed to examine the interrelationships between the TPB determinants and additional variable of perceived risk. Hierarchical multiple regression analyses predicting 1) intentions and 2) behaviour were conducted. For intentions, the TPB variables of attitude, subjective norm, and PBC were entered at Step 1 with perceived risk entered at Step 2. For behaviour, intention and PBC were entered at Step 1 with attitude, subjective norm, and perceived risk entered at Step 2. Subsequently, guidelines as specified by von Haeften, Fishbein, Kasprzyk, and Montano [42] were used to identify the critical beliefs for parents’ intentions to use online information to manage their child’s health care. First, the Pearson product-moment correlation matrix was analysed to identify those beliefs that significantly correlated with parents’ intentions. To identify those beliefs that make independent contributions to intentions, within each belief-based measure, the significant key beliefs were entered in a stepwise multiple regression analysis. Finally, all key beliefs that made an independent contribution to the prediction of intentions were entered into a final regression.

## Results

### Participant characteristics

Participants were 391 parents (*n* = 372 mothers, *n* = 19 fathers) ranging in age from 22 to 67 years (*M* = 34.96 years; *SD* = 5.73). Among the parents, 129 (33%) had one child, 180 (46%) had two children, and 81 (21%) had three or more children. The majority of the parents were in a partnered relationship (*n* = 352, 90%), over half were in paid employment (*n* = 263, 67%), approximately half were university educated (*n* = 217, 55%) and 77 (20%) of the parents had a medical background (e.g., nurse, dietician, physiotherapist), with parents reporting using the internet for an average of 16 hours per week (*SD* = 12.71). Two months later, 181 (46%) of the parents participated in the follow-up (Time 2) questionnaire. A multivariate analysis of variance (*F* (6, 370) = 1.15, *p* = .333) revealed no significant differences on any of the main study constructs assessed in the Main (Time 1) questionnaire between those who did and did not complete both sets of questionnaires. Furthermore, bivariate analyses with Bonferroni adjustment (to avoid chance capitalization) of the underlying beliefs across Time 1 only and Time 1 and 2 respondents revealed no differences, except for the control belief “Having a specific website to look up that has been recommended by others” in which Time 1 only respondents (*M* = 5.87) compared to those that completed both sets of questionnaires (*M* = 6.19) had a significantly lower mean (*t*(389) = -3.22, *p* = .001).

### Descriptive statistics and correlation matrix

The means, standard deviations, and correlations are reported in Table 1. Parents generally had moderate intentions to use online information to manage their child’s health care (*M* = 4.59, *SD* = 1.54), with parents performing this behaviour at a low-to-moderate level in the past 2 months (*M* = 3.20, *SD* = 1.81). Examination of the correlation matrix revealed that intention and behaviour were correlated with all variables with the exception of PBC with behaviour. Attitude correlated the strongest with intention (*r* = .77, *p* < .001) and intention correlated the strongest with behaviour (*r* = .43, *p* < .001).

**Table 1:** **Means, standard deviations, and bivariate correlations for the TPB variables (attitude, subjective norm, PBC), perceived risk, intention (N = 391) and behaviour (N = 181)**

	**Variable**	**1.**	**2.**	**3.**	**4.**	**5.**	**6.**
1.	Attitude		.83***	.30***	-.55***	.77***	.41***
2.	Subjective norm			.41***	-.56**	.74***	.38***
3.	PBC				-.23***	.38***	.14
4.	Perceived risk					-.59***	-.21**
5.	Intention						.43***
6.	Behaviour						
	Mean	5.01	4.69	5.78	3.65	4.59	3.20
	SD	1.29	1.34	1.13	1.54	1.54	1.81

*Note.* Mean scores on 7-point scales (1-7; higher scores stronger agreement, more important). *Note.* PBC = perceived behavioural control.

**p < .01. ***p < .001.

### Regression analysis

A hierarchical multiple regression analysis predicting intentions showed that the Step 1 variables accounted for 68% of the variance in intentions, *F *(3, 375) = 265.24, *p* < .001, with all three TPB predictors (attitude, subjective norm, and PBC) reported as significant. The addition of perceived risk at Step 2 significantly added approximately 2% of the variance, *Fchange* (1, 374) = 18.40, *p* < .001. In the overall model, attitude, subjective norm, PBC, and perceived risk were the significant predictors of parents’ intentions to use online information to manage their child’s health care, *F* (4, 374) = 212.76, *p* < .001 (see Table 2).

**Table 2:** **Hierarchical regression analyses testing the predictors of parents’ intention and behaviour to use child health information from the internet to manage their child’s health**

	**Variable**	**B**	**β**	**95% CI for B**	**R** ^**2**^	**ΔR** ^**2**^
**Prediction of Intentions (N = 379)**						
Step 1	Attitude	.57	.48***	[0.48, 0.66]	.68***	.68***
	Subjective norm	.44	.38***	[0.35, 0.54]		
	PBC	.11	.08*	[0.02, 0.19]		
Step 2						
	Attitude	.51	.43***	[0.420, 0.61]	.70***	.02***
	Subjective norm	.38	.33***	[0.28, 0.48]		
	PBC	.11	.08*	[0.03, 0.19]		
	Perceived risk	-.16	-.15**	[-0.23, -0.08]		
**Prediction of Behaviour (N = 176)**						
Step 1	Intention	.55	.43***	[0.37, 0.72]	.19***	.18***
	PBC	.04	.02	[**-**0.22, 0.29]		
Step 2						
	Intention	.35	.27*	[0.01, 0.68]	.21***	.19***
	PBC	-.01	-.00	[-0.27, 0.25]		
	Attitude	.22	.16	[**-**0.13, 0.58]		
	Subjective norm	.15	.11	[**-**0.16, 0.47]		
	Perceived risk	.10	.08	[-0.12, 0.32]		

Note. PBC = perceived behavioural control; CI = confidence interval; *p < .05. **p < .01. ***p < .001.

An additional regression analysis predicting behaviour was conducted and revealed that intention and PBC entered at Step 1 accounted for 19% of the variance in behaviour, *F* (2, 173) = 20.30, *p* < .001, with intention but not PBC reported as significant. The addition of the Step 2 variables did not significantly explain further variance, *Fchange* (3, 170) = 1.35, *p* = .26. In the overall model, intention was the only significant predictor of parents’ use of online information to manage their child’s health care, *F* (5, 170) = 8.98, *p* < .001 (see Table 2).

### Critical beliefs underlying intention

**Figure 1:**
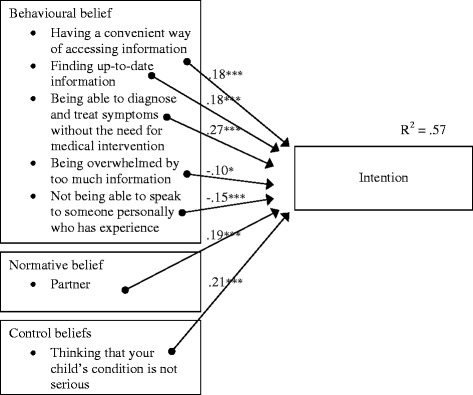
**Critical belief-based targets for parents’ intentions to use internet information to manage their child’s health.** Note: N = 362. R = .75, Adjusted R^2^ = .56, Standard Error of the estimate = 1.02. *p < .05. ***p < .001.

Given that all three TPB variables of attitude, subjective norm, and PBC were found to be significant predictors of intention, their underlying beliefs were analysed to identify the critical beliefs which guide parents’ intentions to use online information to manage their child’s health care. As evidenced in Table 3, individual correlational analyses showed 17 of the 19 behavioural beliefs, all of the normative beliefs, and 4 of the 7 control beliefs were significantly correlated with intention (r = .52 to .10). A regression analysis on the significant behavioural beliefs revealed “having instant access to information” (β = .17), “having a convenient way of accessing information” (β = .13), “finding up-to-date information about my child’s health/development” (β = .22), “being able to diagnose and treat symptoms without the need for medical intervention” (β = .35), “being overwhelmed by too much information” (β = -.11), and “not being able to speak to someone personally who has experience” (β = -.18) as independent contributors to the prediction of intention. A regression analysis on the significant normative beliefs revealed “friends” (β = .27) and “partner” (β = .35) as independent predictors of intention. Regression analysis on the significant control beliefs revealed “having a website address that is easy to remember” (β = .13) and “thinking that your child’s condition is not serious” (β = .37) as predictors of intention. To identify the critical beliefs, the 10 individual belief predictors identified above were entered into a final regression analysis. As shown in Figure 1, in the final model, seven critical beliefs were identified as independently contributing to the prediction of intention, with the final model explaining 57% (adjusted R^2^ = .56) of the variance in parents’ intentions to use child health information from the internet to manage their child’s health care.

**Table 3:** **Means and standard deviations of the individual behavioural, normative, and control beliefs, and correlations with intention (N = 391** ***)***

	**M**	**SD**	**r**
**Beliefs**			
**Behavioural beliefs**			
**Benefits:**			
Having instant access to information	5.88	1.19	.49***
Having a convenient way of accessing information	6.00	1.08	.50***
Using a free or non-costly service	5.67	1.37	.40***
Feeing reassured	5.50	1.30	.40***
Being in control of my child’s health	5.30	1.38	.44***
Having a broad range of information available	5.51	1.39	.37***
Making it easy to access information	5.58	1.39	.37***
Finding extra information about my child’s health/medical condition	6.00	1.04	.39***
Finding up-to-date information about my child’s health/development	5.65	1.28	.48***
Having increased understanding and feeling more informed about my child’s health/medical condition	5.83	1.15	.43***
Being able to diagnose and treat symptoms without the need for medical intervention	3.64	1.70	.51***
**Costs:**			
Being overwhelmed by too much information	3.78	1.60	-.11*
Being uncertain about the trustworthiness of information or its source	4.80	1.50	-.16**
Making a possible misdiagnosis	4.50	1.61	-.16**
Delaying treatment based on the information found when in reality treatment is needed urgently	2.70	1.62	-.20***
Not being able to speak to someone personally who has experience	3.85	1.71	-.22***
Finding out information that causes unnecessary worry or stress	4.46	1.53	-.17**
Finding information that may not be relevant to Australian children	4.30	1.64	-.09
Finding conflicting information	5.29	1.35	-.06
**Normative beliefs**			
Your family members	5.12	1.44	.43***
Your friends	5.33	1.27	.49***
Doctors	3.82	1.56	.24***
Nurses	3.96	1.49	.27***
Your parents	4.76	1.58	.39***
Your mothers’ group/other mothers that you know	5.25	1.35	.39***
Your partner	5.37	1.47	.52***
**Control beliefs**			
**Barriers:**			
Technical issues	3.29	1.94	.10*
Poor website design or content	4.63	1.68	.06
Lack of time to access the internet	3.85	1.89	-.06
Children interfering or interrupting computer/internet access	4.15	1.86	.03
**Motivators:**			
Having a specific website to look up that has been recommended by others	6.02	1.00	.24***
Having a website address that is easy to remember	5.47	1.33	.24***
Thinking that your child’s condition is not serious	5.05	1.62	.39***

*p < .05. **p < .01. ***p < .001.

## Discussion

The current study, grounded in a sound theoretical basis, examined the underlying factors guiding parents’ decisions to use online information to manage their child’s health care, a behaviour which has not yet been explored systematically. We found support for the TPB constructs of attitude, subjective norm, and PBC as well as the additional construct of perceived risk in predicting parents’ intentions, with further support found for intentions, but not PBC, in predicting parents’ behaviour. The results revealed also important information about the critical beliefs that guide parents’ decisions to engage in this child health management behaviour. Table 4 summarises the key findings and potential strategies for interventions that aim to challenge parents’ thoughts about using online information to manage their child’s health care.

**Table 4:** **Summary of the key findings and potential strategies for interventions**

**Factor**	**Intervention focus**	**Critical targets**
Attitude	Dispel perceptions of positive outcomes and address perceptions of negative outcomes	- Convenient way to access information
		- Finding up-to-date information
		- Being able to diagnose and treat symptoms without the need for medical intervention
		- Overwhelmed by too much information
		- Not being able to speak to someone personally who has experience
Subjective norm	Challenge the approval of important others	- Partners
Perceived behavioural control	Address strategies that may encourage use	- Thoughts about the child’s condition not being serious, so use internet rather than seek health professional advice
Perceived risk	Focus on evaluations of the risk involved	- Testimonials from parents and health professionals and evidence from the empirical literature of the potential risks involved in using the internet to access child health information

The results support the TPB in that attitude, subjective norm, and PBC predicted parents’ intentions (Hypothesis 1). These findings suggest that parents who have more favourable attitudes toward using online information to manage their child’s health care, perceive pressure from important referents to engage in this behaviour, and who believe they have higher levels of control regarding their ability to do so, will have stronger intentions to use information from the internet to manage their child’s health care. In the prediction of parents’ behaviour, the TPB was partially supported (Hypothesis 2) as intention, but not PBC, predicted parents’ use of online information to manage their child’s health care. This finding suggests that parents who have stronger intentions to perform the target behaviour are more likely to make decisions to do so.

The finding that PBC did not emerge as a significant predictor of behaviour may be explained by parents’ lack of accuracy in judging how much control they actually have over using information from the internet to manage their child’s health care due to factors outside of their control, such as the information requested not being available or easy to comprehend. According to Ajzen [25], the strength of PBC in determining behaviour is dependent on perceptions of control being reflective of actual control. Additionally, the single-item PBC measure in the current study reflected control rather than self-efficacy and it is suggested that the control component alone may not be the optimal predictor for behaviour as is a general factor of PBC that combines both self-efficacy and controllability [43].

For the additional construct of perceived risk, there was support for Hypothesis 3 in that the construct significantly predicted parents’ intentions. In this case, the less risk perceived by parents in engaging in this behaviour, the more likely they were to use online information to manage their child’s health care. These results support the independent role of risk perceptions in this context and concur with other studies examining the role of risk for people’s decision making [33-35,44,45]. It is likely that the level of perceived risk emanates from the degree of trustworthiness in the information provided online. Those who use online information report trust in the information; this trust, however, is not necessarily immediate as it develops over time as they identify sites matching their social identity, determined through site language, contributions to the site from like-minded people, and gaining a sense of being part of a community [5].

Overall, the psychosocial determinants identified in this study help to understand parents’ decisions to use online information to manage their child’s health care. Specifically, those parents who have a more positive attitude, perceive greater social pressure, believe that have greater control, and perceive lower risks associated with the behaviour, will have stronger intentions to use online information to manage their child’s health care, with stronger intentions predicting behavioural performance.

Given that attitude, subjective norm, and PBC successfully predicted intentions to use information from the internet to manage their child’s health care, this study further investigated those critical beliefs that guide parents’ cognitions to engage in this behaviour (Hypothesis 4). First, the findings of the behavioural beliefs suggest that parents focus on both the positive and negative outcomes of using online information to manage their child’s health care. Specifically, parents believe that the internet provides up-to-date information, and a convenient way of accessing information and a way of diagnosing and treating symptoms without medical intervention. Parents believe also that they may be overwhelmed by the amount of information provided online and that using information from the internet will have the negative consequence of not being able to speak to someone with experience. In challenging these beliefs, a focus needs to be on dispelling perceptions of positive outcomes from using online child health information and addressing perceptions of potential negative outcomes. Parents should be informed of more convenient and reliable ways of accessing up-to-date information to address the problem of too much information online and the types of minor illnesses and/or symptoms that parents could treat without medical interventions. For those wishing to speak to/interact with someone in real time, websites could direct parents to child health help lines or, for those websites hosted or sponsored by larger companies, offer online immediate ‘chat’ assistance to facilitate discussion with a knowledgeable health professional to help with their decision making. Parents should also be made aware that online health information may not always follow current guidelines (e.g., [13]), and that caution needs to be practiced when accessing websites with dated or opinionated information. Appropriate disclaimers about checking information with health professionals should be either mandated or strongly encouraged.

The findings of the normative beliefs suggest that challenging the approval of important others, in particular partner support, for using online information to manage their child’s health care may be warranted in combating inappropriate use of this child health management behaviour. Parents should be encouraged to resist the pressure by one’s partner to use online information to manage their child’s health. Instead, parents could be directed toward established parenting groups as a source of support. Parenting groups provide a social network that can help to increase parents’ satisfaction and exchange health information [46]. These groups could be provided with up-to-date websites of evidence-based health information that members of the group can access which will enable group members to have reliable health information that can then be shared with partners and friends.

Health professionals can also play an important role in helping parents resist pressure from important others to use online health information to manage their child’s health. Doctors and child health nurses, for example, during the consultation process can take the opportunity to educate parents about online health information and provide parents with evidenced-based health information for treating common childhood conditions.

In addition, the findings of the control beliefs suggest that performing the target behaviour is facilitated by parents thinking that their child’s condition is not serious. This belief, coupled with the positive outcome belief of being able to diagnose and treat symptoms without the need for medical intervention could potentially lead to undesired consequences for the health of the child, especially if the child is misdiagnosed by the parent and actually in need of medical attention [18]. As such, testimonials from parents and evidence from the empirical literature of the consequences of a misdiagnosis based on online information on health outcomes may facilitate parents to question their use of internet-based information to manage their child’s health care.

While the results of the current study are the first to provide valuable information grounded in sound theory to help understand parents’ decisions to use online information to manage their child’s health care, the findings should be interpreted in light of the study’s limitations. No information was collected on the context of the family situation or child health status to determine why parents would be in the position to be searching online for health information. However, we did ask about a wide range of possible information that could be searched online and for which could cover many situations, not just for a health crisis or condition, for both the parent and child (e.g., appropriate age to introduce solids, vaccination). Furthermore, the 2 month follow up may not have been a long enough time period for which circumstances would arise where parents are then motivated to search online for health information. Additional limitations include the over representation of mothers and the use of self-report measures of behaviour and 1-item scales for the TPB and additional variables to reduce the length of the questionnaire for time-pressed parents. Further research is required to validate the results from the present study for mothers and carers (including a greater number of fathers and extended family), preferably with multiple-item scales. Furthermore systematic validation of behaviour should occur preferably against some other data that monitors or tracks what parents are doing online (e.g., via a diary). Finally, other potentially important factors such as the role of motivations, planning, and social influences [47,48] have been known to impact on people’s decision making. Thus, it may be useful for future research to investigate additional, theoretically relevant variables in this context to determine if they exert any further explanatory power.

## Conclusions

Overall, we found support for the efficacy of the TPB and the role of perceived risk in understanding parents’ decisions for using online information to manage their child’s health care. Challenging parents’ attitudes and beliefs toward this child health care management behaviour is important given the increase in internet usage and the questionable quality of health information provided online. This theory-based investigation to understand parents’ motivations and online information-seeking behaviour can assist in the development of recommendations and policies to guide more appropriate help-seeking actions among parents.

## Additional files

Additional file 1:
**Using the internet for child health information main questionnaire.**
Additional file 2:
**Using the internet for child health information follow-up questionnaire.**
