# Supplementary material for: Use of online health information to manage children’s health care: a prospective study investigating parental decisions
Source: BMC Health Serv Res. 2015 Apr 2;15:131. doi: 10.1186/s12913-015-0793-4 (PMC4392618; doi:10.1186/s12913-015-0793-4)
Supplement: Additional file 1: — Using the internet for child health information main questionnaire. [file 12913_2015_793_MOESM1_ESM.docx]

| 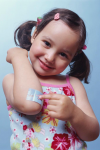 | **USING THE INTERNET FOR CHILD HEALTH INFORMATION SURVEY** | 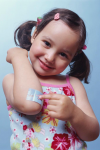 |
| --- | --- | --- |

**Thank you for participating in this study**

All the information you provide us will be anonymous and confidential. To assist us with the processing of data, your survey responses will be identified only by an anonymous code.

**Please be sure to follow the instructions below to create your anonymous code**.

| **Your participant code identifier** |
| --- |

|  | **What is the first letter of your first name?** |
| --- | --- |
|  | **What is the third letter of your first name?** |
|  | **What is the first letter in your mother’s first name?** |
|  | **What is the day of the month you were born on?** (e.g., 24th) |

**For example, imagine Louise Smith is filling out the questionnaire. Her mother’s name is Anne. Louise was born on the 31^st^ of December. Her code identifier would be:**

**L**

**U**

**A**

**31**

| **PART 1 - Please tell us a little bit about yourself...** |
| --- |

1. What is your **postcode**? __________
2. Your **age** at your last birthday (please specify): ________ years
3. Your **gender** (please choose one):

□ Male

□ Female

1. Your **marital status** (please choose one):

□ Single (never married)

□ In a relationship

□ Married

□ Defacto

□ Divorced/Separated

□ Widowed

1. The **number of children** you have (please specify): _______
2. Please write the **date of birth for each of your children** (e.g., 15/12/2005) (please specify):

Birth date of 1^st^ child: ___________________

Birth date of 2^nd^ child: __________________

Birth date of 3^rd^ child: __________________

Birth date of 4^th^ child: __________________

Birth date of 5^th^ child: __________________

Birth date of 6^th^ child: __________________

1. What is your **current occupation** (please specify): ________________________
2. Are you **currently in paid employment** (please choose one):

□ Yes

□ No (**please go to question 9**)

□ On leave

1. If **YES**, is this **employment** (please choose one):

□ Full time

□ Part time

□ Casual

1. Please choose the **highest level of education** you have **completed**:

□ Primary school

□ Secondary school

□ TAFE course

□ University degree

□ Postgraduate degree

□ Currently studying (please specify): _______________________________

1. The **type of internet connection you have at home** is:

□ Broadband Mobile

□ Broadband

□ ADSL

□ Dial up

□ Other (please specify): ________________________________________

1. Do you have **internet access at work** (please choose one)?

□ Yes

□ No

1. Approximately **how many hours a week overall would you use the internet** (please specify)? ______________ hours
2. Do you **use the internet t**o search for information **to care for your** **own health**?

□ Yes

□ No

1. Do you **use the internet** to search for information **to care for your** **child’s health**?

□ Yes

□ No

1. Would you be **comfortable sharing with your doctor** or other medical professional that you **obtained information from the internet**?

□ Yes

□ No

□ Unsure

| **PART 2 - Using child health information from the internet to MANAGE YOUR CHILD’S HEALTH** |
| --- |

We acknowledge that there are many different approaches a parent may take in caring for their child/ren. There are no right or wrong answers. We are interested purely in YOUR opinions. If you are unsure how to answer a question please give the best answer you can.

The following questions relate to your beliefs about **using child health information from the internet to manage your child’s health care** in the next 2 months.

When we talk about **child health information** we mean any **information that you may find online that helps you to make a decision about how to care for your child’s health**.

This information may include, but is not limited to, information about:

- the appropriate age to introduce solids
- management of an existing condition such as asthma or dietary intolerances
- vaccination
- an upcoming medical procedure or test
- advice about how to identify; diagnose, or treat a rash or a fever
- administer first aid
- manage allergic reactions
- identify side effects of a prescribed medication
- any other information that you may use to make a decision about your child’s health care.

| If you were concerned about your child’s health in the next 2 months, how **likely** is it that **using child health information from the internet** to manage your child’s health care would **result** in the following?  Please circle one option on **each** line | **Extremely unlikely** | **Very unlikely** | **Slightly unlikely** | **Neither likely nor unlikely** | **Slightly likely** | **Very likely** | **Extremely likely** |
| --- | --- | --- | --- | --- | --- | --- | --- |
| Having instant access to information | 1 | 2 | 3 | 4 | 5 | 6 | 7 |
| Making it easy to access information (e.g., portable, rural access, accessible when doctor is not available) | 1 | 2 | 3 | 4 | 5 | 6 | 7 |
| Having a convenient way of accessing information (e.g., can process information in own time, in the comfort of own home, can refer back to information at a later time) | 1 | 2 | 3 | 4 | 5 | 6 | 7 |
| Using a free or non-costly service | 1 | 2 | 3 | 4 | 5 | 6 | 7 |
| Feeling reassured (e.g., confirms information that you have been given by a doctor) | 1 | 2 | 3 | 4 | 5 | 6 | 7 |
| Being in control of my child’s health | 1 | 2 | 3 | 4 | 5 | 6 | 7 |
| Having a broad range of information available (e.g., different points of view, other people’s experiences, online forums etc) | 1 | 2 | 3 | 4 | 5 | 6 | 7 |
| Finding extra information about my child’s health/medical condition (e.g., pictures, terminology, treatment options, symptoms, side effects of medication, etc) | 1 | 2 | 3 | 4 | 5 | 6 | 7 |
| Finding up to date information about my child’s health/development | 1 | 2 | 3 | 4 | 5 | 6 | 7 |
| Having increased understanding and feeling more informed about my child’s health/medical condition (e.g., learning something to ask the doctor, finding information in lay persons terms) | 1 | 2 | 3 | 4 | 5 | 6 | 7 |
| Being able to diagnose and treat symptoms without the need for medical intervention | 1 | 2 | 3 | 4 | 5 | 6 | 7 |
| Being overwhelmed by too much information | 1 | 2 | 3 | 4 | 5 | 6 | 7 |
| Being uncertain about the trustworthiness of information or its source (e.g., inaccurate information etc) | 1 | 2 | 3 | 4 | 5 | 6 | 7 |
| Making a possible misdiagnosis | 1 | 2 | 3 | 4 | 5 | 6 | 7 |
| Delaying treatment based on the information found when in reality treatment is needed urgently | 1 | 2 | 3 | 4 | 5 | 6 | 7 |
| Not being able to speak to someone personally who has experience | 1 | 2 | 3 | 4 | 5 | 6 | 7 |
| Finding out information that causes unnecessary worry or stress | 1 | 2 | 3 | 4 | 5 | 6 | 7 |
| Finding information that may not be relevant to Australian children | 1 | 2 | 3 | 4 | 5 | 6 | 7 |
| Finding conflicting information | 1 | 2 | 3 | 4 | 5 | 6 | 7 |

| If you were concerned about your child’s health in the next 2 months, how **likely** is it that the following **people or groups** would **approve** of or support your **use of child health information from the internet** to manage your child’s health care?  Please circle one option on **each** line | **Extremely unlikely** | **Very unlikely** | **Slightly unlikely** | **Neither likely nor unlikely** | **Slightly likely** | **Very likely** | **Extremely likely** | **Doesn’t apply to me** |
| --- | --- | --- | --- | --- | --- | --- | --- | --- |
| Your family members | 1 | 2 | 3 | 4 | 5 | 6 | 7 | 8 |
| Your friends | 1 | 2 | 3 | 4 | 5 | 6 | 7 | 8 |
| Doctors | 1 | 2 | 3 | 4 | 5 | 6 | 7 | 8 |
| Nurses (e.g., child health nurse) | 1 | 2 | 3 | 4 | 5 | 6 | 7 | 8 |
| Your Parents | 1 | 2 | 3 | 4 | 5 | 6 | 7 | 8 |
| Your mother’s group/other mothers that you know | 1 | 2 | 3 | 4 | 5 | 6 | 7 | 8 |
| Your partner | 1 | 2 | 3 | 4 | 5 | 6 | 7 | 8 |

| If you were concerned about your child’s health in the next 2 months, how **likely** are the following factors to **prevent** you from **using child health information from the internet** to manage your child’s health care?  Please circle one option on **each** line | **Extremely unlikely** | **Very unlikely** | **Slightly unlikely** | **Neither likely nor unlikely** | **Slightly likely** | **Very likely** | **Extremely likely** |
| --- | --- | --- | --- | --- | --- | --- | --- |
| Technical issues (e.g., computer or internet too slow, not working, blackout etc) | 1 | 2 | 3 | 4 | 5 | 6 | 7 |
| Poor website design or content (e.g., too much advertising, website difficult to navigate) | 1 | 2 | 3 | 4 | 5 | 6 | 7 |
| Lack of time to access the internet | 1 | 2 | 3 | 4 | 5 | 6 | 7 |
| Children interfering or interrupting computer/internet access | 1 | 2 | 3 | 4 | 5 | 6 | 7 |

| If you were concerned about your child’s health in the next 2 months, how **likely** are the following factors to **encourage** you to **use child health information from the internet** to manage your child’s health care?  Please circle one option on **each** line | **Extremely unlikely** | **Very unlikely** | **Slightly unlikely** | **Neither likely nor unlikely** | **Slightly likely** | **Very likely** | **Extremely likely** |
| --- | --- | --- | --- | --- | --- | --- | --- |
| Having a specific website to look up that has been recommended by others (e.g., doctor, friend, child health organisation, government etc) | 1 | 2 | 3 | 4 | 5 | 6 | 7 |
| Having a website address that is easy to remember | 1 | 2 | 3 | 4 | 5 | 6 | 7 |
| Thinking that your child’s condition is not serious (so more inclined to use the internet than seek advice from a health professional such as a doctor) | 1 | 2 | 3 | 4 | 5 | 6 | 7 |

If you were concerned about your child’s health in the next 2 months, how much do you agree with the following statements about **using child health information from the internet to manage your child’s health care**? (Please circle a number on each line)...

|  | **Strongly disagree** | **Disagree** | **Somewhat disagree** | **Neither agree nor disagree** | **Somewhat agree** | **Agree** | **Strongly agree** | **Doesn’t apply to me** |
| --- | --- | --- | --- | --- | --- | --- | --- | --- |
| **Using** child health information from the **internet** to **manage my child’s health care** would be **good** | 1 | 2 | 3 | 4 | 5 | 6 | 7 |  |
| I **intend** to **use** child health information from the **internet** to **manage my child’s health care** | 1 | 2 | 3 | 4 | 5 | 6 | 7 |  |
| Most **people who are important to me** would **support/approve** of me **using** child health information from the **internet** to **manage my child’s health care** | 1 | 2 | 3 | 4 | 5 | 6 | 7 |  |
| It is **mostly up to me** whether I **use** child health information from the **internet** to **manage my child’s health care** | 1 | 2 | 3 | 4 | 5 | 6 | 7 |  |
| If I **used** child health information from the **internet** to **manage my child’s health care**, I **would regret it** | 1 | 2 | 3 | 4 | 5 | 6 | 7 |  |
| **Most other mothers that I know** **use** child health information from the **internet** to **manage their child’s health care** | 1 | 2 | 3 | 4 | 5 | 6 | 7 | 8 |
| It would be **risky for me** to **use** child health information from the **internet** to **manage my child’s health care** | 1 | 2 | 3 | 4 | 5 | 6 | 7 |  |
| In the **past two months,** **I have** **used** child health information from the **internet** to **manage my child’s health care** | 1 | 2 | 3 | 4 | 5 | 6 | 7 |  |

That was the end of the survey.

Thank you very much for your time ☺

**PLEASE TURN TO THE NEXT PAGE**

If you would be willing to be recontacted in one month’s time to complete a 5 minute follow up survey via telephone or email, please list your contact details below:

Contact preference: Phone / Email

Phone number: ____________________________________

Best time/s to call: __________________________________

Email address: _____________________________________

Thank you again, your help is much appreciated.

**PLEASE TURN TO THE NEXT PAGE**

To thank you for your valuable time taken to complete this survey we are offering you the chance to win one of 5 $50 Coles gift cards. If you would like to be entered into the prize draw, please list your contact details below:

Contact preference: Phone / Email

Phone number: ____________________________________

Email address: ____________________________________

Thank you again, your help is much appreciated.
