# Supplementary material for: Use of online health information to manage children’s health care: a prospective study investigating parental decisions
Source: BMC Health Serv Res. 2015 Apr 2;15:131. doi: 10.1186/s12913-015-0793-4 (PMC4392618; doi:10.1186/s12913-015-0793-4)
Supplement: Additional file 2: — Using the internet for child health information follow-up questionnaire. [file 12913_2015_793_MOESM2_ESM.docx]

**CHILD HEALTH INFORMATION FOLLOW UP SURVEY**

| **Your participant code identifier** |
| --- |

|  | **What is the first letter of your first name?** |
| --- | --- |
|  | **What is the third letter of your first name?** |
|  | **What is the first letter in your mother’s first name?** |
|  | **What is the day of the month you were born on?** (e.g., 24th) |

**For example, imagine Louise Smith is filling out the questionnaire. Her mother’s name is Anne. Louise was born on the 31^st^ of December. Her code identifier would be: LUA31**

**PART 1 - Please tell us a little bit about yourself...**

1. What is your **postcode**? _________________
2. Your **age** at your last birthday (please specify): ________ years
3. Your **gender** (please choose one): □ Male □ Female
4. Your **marital status** (please choose one):

□ Single (never married) □ In a relationship □ Married

□ Defacto □ Divorced/Separated □ Widowed

1. The **number of children** you have (please specify): _______
2. Approximately **how many hours a week overall** in the past 2 months **did you use the internet** (please specify)? ______________ hours
3. In the past 2 months, did you **use the internet t**o search for information **to care for your** **own health**? □ Yes □ No
4. In the past 2 months, did you **use the internet** to search for information **to care for your** **child’s health**? □ Yes □ No
5. If YES, **approximately how many times** in the past 2 months did you use the internet to search for information to care for your child’s health? _________

**PLEASE TURN TO THE NEXT PAGE**

**PART 2 – Your USE OF THE INTERNET FOR CHILD HEALTH INFORMATION in the past 2 months**

The following questions relate to your **use of child health information from the internet to manage your child’s health care** in the past 2 months.

When we talk about **child health information** we mean any **information that you may have found online that helped you to make a decision about how to care for your child’s health**.

This information may have included, but is not limited to, information about:

- the appropriate age to introduce solids
- management of an existing condition such as asthma or dietary intolerances
- vaccination
- an upcoming medical procedure or test
- advice about how to identify; diagnose, or treat a rash or a fever
- administer first aid
- manage allergic reactions
- identify side effects of a prescribed medication
- any other information that you may use to make a decision about your child’s health care.

**How do you agree with the following statements** (please select a response on each line)?

|  | **Strongly disagree** | **Disagree** | **Somewhat disagree** | **Neither agree nor disagree** | **Somewhat agree** | **Agree** | **Strongly agree** |
| --- | --- | --- | --- | --- | --- | --- | --- |
| In the **past two months,** **I have** **used** child health information from the **internet** to **manage my child’s health care** | 1 | 2 | 3 | 4 | 5 | 6 | 7 |

**PLEASE TURN TO THE NEXT PAGE**

**Please respond to the following statements** (please select a response on each line)?

|  | **Never** | **Rarely** | **Sometimes** | **About half the time** | **Often** | **Most of the time** | **Always** |
| --- | --- | --- | --- | --- | --- | --- | --- |
| In the **past two months,** **how often** did you use child health information from the **internet** to **manage your child’s health care** | 1 | 2 | 3 | 4 | 5 | 6 | 7 |

That was the end of the survey.

Thank you very much for your time ☺

**Thank you again; your help is much appreciated**
